# A multiple-shape memory polymer-metal composite actuator capable of programmable control, creating complex 3D motion of bending, twisting, and oscillation

**DOI:** 10.1038/srep24462

**Published:** 2016-04-15

**Authors:** Qi Shen, Sarah Trabia, Tyler Stalbaum, Viljar Palmre, Kwang Kim, Il-Kwon Oh

**Affiliations:** 1 Active Materials and Smart Living Laboratory, Department of Mechanical Engineering, University of Nevada, Las Vegas (UNLV), Las Vegas, NV, 89154, USA; 2 Department of Neurosurgery, The University of Texas Health Science Center at Houston, Medical School, Houston, TX 77030, USA; 3 Creative Research Initiative Center for Functionally Antagonistic Nano-Engineering, Department of Mechanical Engineering, Korea Advanced Institute of Science and Technology (KAIST), Daejeon 34141, Republic of Korea

## Abstract

Development of biomimetic actuators has been an essential motivation in the study of smart materials. However, few materials are capable of controlling complex twisting and bending deformations simultaneously or separately using a dynamic control system. Here, we report an ionic polymer-metal composite actuator having multiple-shape memory effect, and is able to perform complex motion by two external inputs, electrical and thermal. Prior to the development of this type of actuator, this capability only could be realized with existing actuator technologies by using multiple actuators or another robotic system. This paper introduces a soft multiple-shape-memory polymer-metal composite (MSMPMC) actuator having multiple degrees-of-freedom that demonstrates high maneuverability when controlled by two external inputs, electrical and thermal. These multiple inputs allow for complex motions that are routine in nature, but that would be otherwise difficult to obtain with a single actuator. To the best of the authors’ knowledge, this MSMPMC actuator is the first solitary actuator capable of multiple-input control and the resulting deformability and maneuverability.

Many life forms have unique organs that exhibit high deformability, maneuverability, and control. For example, most fish have a pectoral fin that can bend and twist in different ways to create various swimming motions, such as hovering and flapping^1^,^2^,^3^. Biomimicry has been a great inspiration to scientists and engineers. Materials and robotic systems have been developed over the last 25 years to reproduce the motion and abilities of life forms as best as possible^4^,^5^,^6^,^7^,^8^,^9^,^10^,^11^. Various projects have been able to demonstrate the ability to mimic these natural soft tissue systems, although only to a limited extent in terms of control and simplicity^12^,^13^,^14^.

One common method to develop a multiple degrees-of-freedom actuator is to use mechanisms. Recently, robotics has emerged that are driven by hydraulic and pneumatic pressure. Marchese *et al.*^15^ reported a soft-body robotic fish that can swim in three dimensions with its hydraulic actuator, which was driven by a gear pump. Tolley *et al.* developed a pneumatically powered soft robot with four flexible legs^16^. The robot could move under various conditions of terrain. Inspired by an elephant trunk, an actuator was developed for assistance in object handling^17^. This flexible robot, which was actuated pneumatically, could perform complicated deformations. On the other hand, many robotic systems with motor-driven actuators have been developed. For example, robotic arms with actuation having multiple degrees-of-freedoms have been studied for decades; these robotic arms could be driven by cables^18^ or directly by motors^19^. Most actuators contain mechanisms that have large size and complex structures. Hence, their application is limited with regard to small-sized robots. Moreover, undesired motor noise could be produced when operating the system.

Major contributions to these studies have been made on smart materials, such as shape memory alloys (SMA), shape memory polymers (SMP), piezoelectric materials, ionic polymer actuators, and other artificial muscle devices and systems^20^,^21^,^22^,^23^,^24^,^25^,^26^,^27^,^28^. Another method to achieve complex deformation is to use the shape memory effects of polymers and alloys. SMPs and SMAs can recover their original shapes by means of external stimuli, such as thermal or electrical inputs^29^,^30^. Besides shape changing, however, most robotic systems require reciprocating motion, which enables them to propel themselves forward. Most SMPs respond to thermal change. They recover their shape over a relatively long period, and cannot respond instantaneously. This limits SMPs to be applied only for shape change; they cannot be used as fast dynamic actuators. A recent application example of SMPs involved a crawling robot with an origami structure, developed by Felton *et al.*^31^. The shape memory composites were utilized only during the folding process. Complex electronics were embedded to achieve the crawling motion, and the overall motion was driven by motors. Shape-memory effects also have been reported in ionic polymer materials, such as Nafion™. Xie presented a highly-tunable method of creating multiple shape-memory effects in an individual Nafion™ sample by fixing different shapes at several temperatures^32^. Rossiter *et al.* showed that actuators using a Nafion™-based ionic polymer-metal composite (IPMC) have shape-memory effects^33^.

Another method involves using electro-mechanical transitions of smart materials, such as piezoelectric actuators or electroactive polymer actuators. Piezoelectric materials have been widely used as actuators on micro-robots, such as swimming and flying robots^20^. Many actuators have been developed using electroactive polymers. In particular, robots have been developed with ionic polymer-metal composites (IPMCs) as actuators and sensors^34^,^35^,^36^,^37^,^38^,^39^,^40^. For example, Palmre *et al.* developed a soft bio-inspired fin^41^. Complex deformation can be obtained by integrating IPMC actuators and by selectively actuating. However, most of the actuators perform a bending/oscillating motion near a neutral position. Maintaining a static position other than neutral due to hysteresis and creep in IPMCs^24^ has been shown to be difficult. To date, no single actuator has been capable of dynamic control of complex twisting and bending deformations, either simultaneously or separately. For example, SMP actuators can exhibit complex deformation by means of electric or thermal inputs, but they cannot achieve multiple-input control simultaneously for independent control of twisting and bending motions^42^,^43^.

This study introduces a multiple degrees-of-freedom soft multiple shape-memory polymer-metal composite (MSMPMC) actuator. Similar to the structure of IPMC actuators, the MSMPMC is composed of two or more electrodes separated by an ion-conductive polymer material (Fig. 1a–c). Under an applied voltage, the transport of ions and water molecules as well as the associated electrostatic interactions within the polymer result in a bending deformation, which is the electro-mechanical actuation effect (Fig. 1d,e)^44^. The electro-mechanical actuation effect has the capabilities of resilience, inherent softness, and biocompatibility.

Shape memory polymers are materials that can memorize a permanent shape, and then later return to their original shape under specific conditions of external thermal, electrical, or other stimulation^45^,^46^. They have the advantages of high elastic deformation, low cost, low density, and potential biocompatibility and biodegradability. Nafion™, perfluorinated alkene with short side-chains terminated by ionic group of sulfonate, was shown to be able to ‘memorize’ multiple shapes under multiple temperature programming, which is the multiple shape-memory effect^32^. The Nafion™ has a broad glass transition temperature, which is from ~55 °C to ~130 °C. Assuming the original shape of Nafion™ is S0. When the Nafion™ is fixed with an extra load in shape S1, heated to programming temperature T_p1_ for three minutes and cooled to fixing temperature T_f1_ for one minute, the shape S1 is ‘memorized’ within the temperature range from T_p1_ to T_f1_. Upon reheating the Nafion™ to T_p1_, the Nafion™ can recover to shape S1. The broad glass transition can be divided into a series of individual glass switching transitions for each programmed shape. Different shapes S1, S2, S3 are heated to programming temperatures T_p1_, T_p2_, T_p3_ and cooled to T_f1_, T_f2_, T_f3_ respectively, where T_p1_ > T_f1_ > T_p2_ > T_f2_ > T_p3_ > T_f3_. Multiple shapes S1, S2 and S3 are programmed at each individual temperature range T_p1_ ~ T_f1_, T_p2_ ~ T_f2_, T_p3_ ~ T_f3_. When the Nafion™ is reheated from T_f3_ to T_p1_, multiple shapes recovered at each temperature range through glass transition. The crystalline segments, which work as physical crosslinks, hold the programmed shapes during each glass transition. This multiple shape memory effect cannot be repeated. The multiple shape memory effect of a Nafion™ fiber is demonstrated in Fig. 2. Since it is controlled by the thermal input, the multiple shape memory effect can also be seen as thermo-mechanical actuation effect.

Based on these two effects, which are electro-mechanical and thermo-mechanical actuation effect, the MSMPMC can perform deformation with multiple degrees of freedom. Several shapes can be programmed into MSMPMC material memory at various temperatures, which enables thermo-mechanical actuation effect. This type of actuator demonstrates high maneuverability by controlling two external inputs – electrical input and thermal input - allowing the complex twisting, bending, and oscillating motions that are frequently observed in nature-made systems.

*Through the electro-mechanical actuation effect*, the actuator is able to perform high-frequency bending motions under external electrical input. *With the thermo-mechanical actuation effect*, the actuator can obtain stable, complex motion under external thermal inputs. Compared with the electro-mechanical actuation effect, the thermos-mechanical actuation effect occurs over a much longer timescale. The ability to control MSMPMC actuators by two external inputs, electrical and thermal, enables these devices to be used to perform highly complex motions, twisting, bending and oscillating simultaneously or separately. The twisting and bending motions are induced thermally and the oscillating motion is induced electrically. The bending motion and oscillating motion take place with the same rotation axis; previously, this could be realized only with existing actuator technologies by using multiple actuators or another complicated robotic system. Moreover, to the best of the authors’ knowledge, the MSMPMC actuator presented in this paper is the first solitary actuator capable of multiple-input control and the resulting deformability and maneuverability.

## Results

### Shape programming of MSMPMC

**Figure 3:**
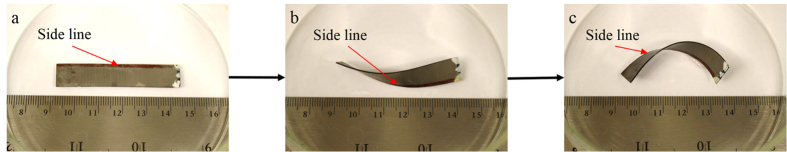
Programming of MSMPMC. (**a**) The original shape of MSMPMC. The length, width, and thickness of the MSMPMC were 51.81 mm, 10.49 mm, and 0.60 mm, respectively. The tip of the MSMPMC was painted white to facilitate image analysis. A side line was painted on the MSMPMC to distinguish the deformation. (**b**) The first shape of the MSMPMC was programmed by heating at 85 °C and cooling at 70 °C. The MSMPMC was wrapped around a rod during the programming. (**c**) The second shape of the MSMPMC was programmed by heating at 60 °C and cooling at 22 °C.

The MSMPMC sample is shown in Fig. 3a. The length, width, and thickness of the MSMPMC are 51.81 mm, 10.49 mm, and 0.60 mm, respectively. The desired twisted shape of the MSMPMC was programmed prior to actuation. The broad glass transition temperature of the Nafion™ is ~55 °C to ~130 °C. The MSMPMC would be programmed and tested in deionized water and the boiling temperature of the water is 100 °C. Thus, the programming temperature of MSMPMC would be ~55 °C to ~100 °C. To program the shapes into the MSMPMC, it was wrapped around a metal rod and fixed. By heating to programming temperature *T*_1_ for three minutes and cooling to fixing temperature *T*_2_ for one minute, the shape was ‘memorized’ within the temperature *T*_1_ to *T*_2_. When the MSMPMC was reheated to *T*_2_, it would recover to the programmed shape through glass transition. Dual shapes were programmed using this process. For distancing the two glass transitions, the programming temperatures were chosen to be 85 °C, 60 °C and the fixing temperatures were chosen to be 70 °C and 22 °C, respectively. To program the first shape, the MSMPMC was heated to 85 °C for three minutes and cooled to 70 °C for one minute (Fig. 3b) in deionized water. Then, the MSMPMC was deformed on the other side further using the same metal rod. To program the second shape, the MSMPMC was heated to 60 °C for three minutes and cooled to 22 °C for one minute (Fig. 3c) in deionized water.

### Deformation analysis of MSMPMC

**Figure 4:**
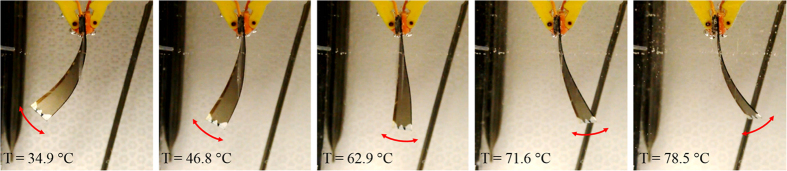
A MSMPMC actuator with multiple degree-of-freedom deformation. The sample was under a sinusoid AC voltage of 3.7 V initial amplitude and 1 Hz frequency. The water was heated from 22 °C (room temperature) to 90 °C.

Figure 4 shows the sequential photographs of an MSMPMC actuator in deionized water. A sinusoid AC voltage of 3.7 V initial amplitude and 1 Hz frequency was applied to the MSMPMC as external electrical input. An immersion heater was used to heat the deionized water as an external thermal input. To cover the programming temperature range, the water was heated from room temperature (22 °C) to 90 °C. As the water temperature increased, the MSMPMC gradually recovered to its previously programmed shapes.

During the experiments, oscillation of the actuator was noticed under the applied voltage. The MSMPMC gradually bent from the left side to the right side, as observed from the front view, and twisted in the clockwise direction, as observed from the top view. To measure the 3D deformation of the actuator, two cameras were used for recording, and image analysis was conducted. The tip of the MSMPMC was painted white to facilitate image analysis. Three points were tracked at two corners and the middle of the actuator tip. In general, the MSMPMC actuator performed twisting, bending, and oscillating motions simultaneously.

**Figure 5:**
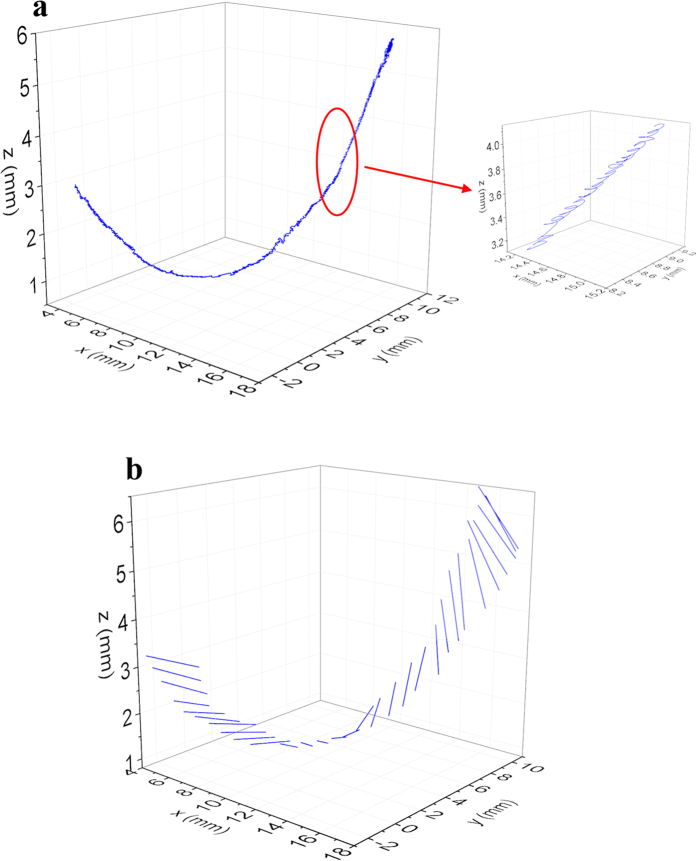
MSMPMC 3D motion trajectory. (**a**) 3D position track of MSMPMC actuator. The applied sinusoid AC voltage has 3.7 V initial amplitude and 1 Hz frequency. The measured temperature increased from 34.9 °C to 84.3 °C. (**b**) 3D motion of the MSMPMC tip line.

Figure 5a shows the 3D track of MSMPMC in the middle of the tip. It was readily observable that the actuator deformed in a twisting and bending motion, resulting in a spiral motion of the tip. This was because the oscillation motion of the MSMPMC was perpendicular to the surface. Since the actuator was twisted and bended, it oscillated in 3D directions along its length, which resulted in the spiral motion of the tip. Figure 5b shows the 3D motion of the MSMPMC tip position, including the bending and twisting motion.

**Figure 6:**
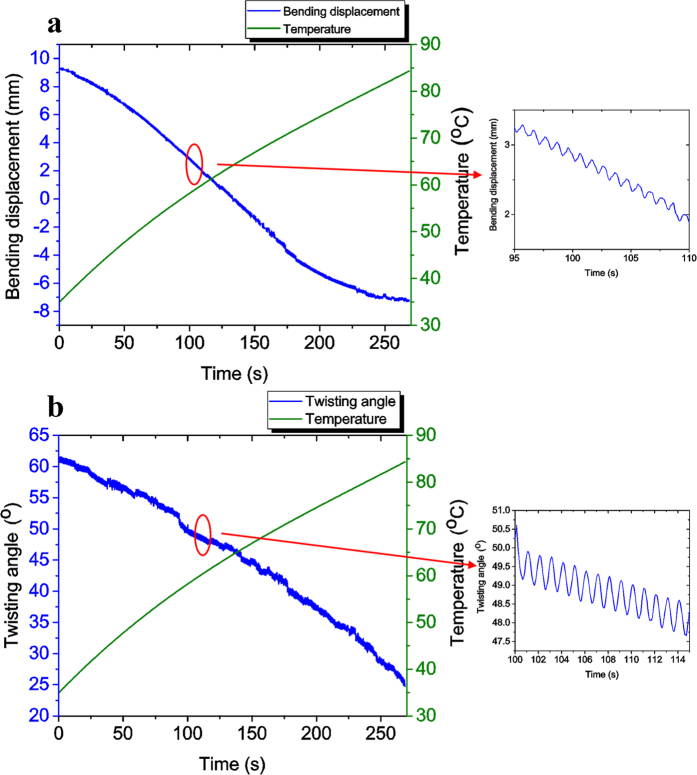
Deformation of MSMPMC. (**a**) Bending displacement and temperature of MSMPMC versus time under an external electrical input of 3.7 V initial amplitude and 1 Hz frequency and thermal input from 34.9 °C to 84.3 °C. (**b**) Twisting angle and temperature of MSMPMC versus time under external electrical and thermal input.

Based on the results shown in Fig. 5, the bending motion and twisting motion were analyzed separately. Figure 6a shows the corresponding bending displacement of the MSMPMC. The bending displacement was obtained by calculating the displacement of the middle tip line point in the orthogonal direction. It can be seen that the MSMPMC performed a gradual but large bending motion, which resulted from thermal actuation via the thermo-mechanical actuation. This motion was combined with a higher-frequency, lower-displacement sinusoidal oscillation, which resulted from electro-mechanical actuation. From the thermal actuation aspect, a total bending displacement of 16.6 mm was measured with the temperature increasing from 34.9 °C to 84.3 °C. The time of the total bending is 268.6 s; From the electrical actuation aspect, the oscillation amplitude is approximately 0.13 mm in the orthogonal direction and the oscillation frequency is 1 Hz.

Along with the bending motion, a twisting motion was performed by the MSMPMC. Figure 6b shows the twisting deformation of MSMPMC. The twisting angle was obtained by calculating the angle difference between the two end lines of the MSMPMC. As the temperature increased from 34.9 °C to 84.3 °C, the MSMPMC twisted by 36.6° due to the thermo-mechanical actuation. As the MSMPMC twisted, the electro-mechanical actuation effect resulted in an oscillation.

### Electrical analysis of MSMPMC

**Figure 7:**
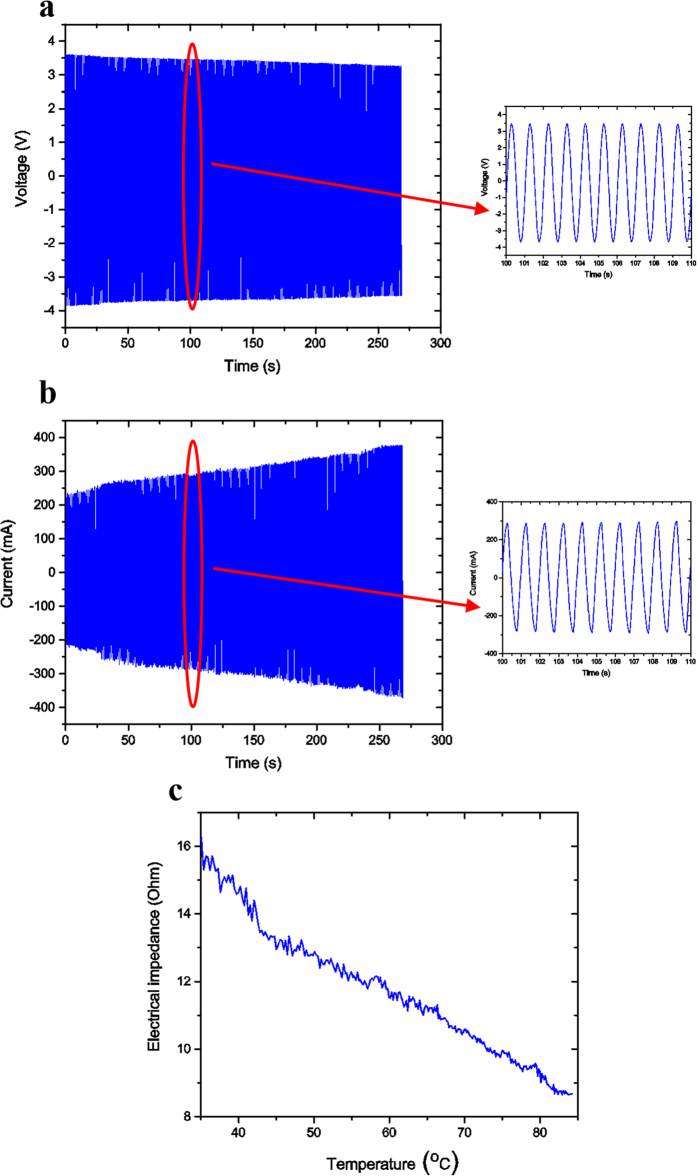
Impedance response of MSMPMC. (**a**) Measured voltage response of MSMPMC versus time. The initial amplitude of sinusoid voltage input was 3.7 V and the frequency was 1 Hz. (**b**) Measured current response of MSMPMC versus time. (**c**) Electrical impedance of MSMPMC versus temperature.

Figure 7a,b show the applied voltage and output current of the MSMPMC during the experiments. A sinusoid voltage signal with an amplitude of 3.7 V and frequency of 1 Hz was applied to the MSMPMC. The MSMPMC was connected in series with a resistor in the circuit. It is interesting to note that as the temperature increased, the measured voltage response of the MSMPMC decreased from 3.7 V amplitude to 3.3 V amplitude. The voltage amplitude decreased by 10.8%. Meanwhile, the current increased from 217 mA amplitude to 374 mA amplitude. The current amplitude increased by 72%. One possible reason is that with the increasing temperature of the MSMPMC (see Fig. 6), the resistance of the surface electrode decreased and the movement of the ion in the polymer increased. As a result, the total electrical impedance of the MSMPMC decreased and the total electrical resistance of the circuit decreased. The current of the circuit increased and the voltage on the MSMPMC decreased. Figure 7c show the electrical impedance of MSMPMC. It can be seen that the electrical impedance shows an overall decrease from 16.3 Ω to 8.7 Ω as the temperature increases. The mechanical impedance change of MSMPMC has yet to be studied.

In addition, experiments were conducted to obtain the fixity and recovery rates of the MSMPMC. The MSMPMC was bended by wrapping around a cylinder, namely the fixed shape. The programming process is the same as previous. The first programmed shape *S*_1_ was programmed by heating to 85 °C and cooling to 70 °C. The second programmed shape *S*_2_ was programmed by heating to 60 °C and cooling to 22 °C. The MSMPMC recovered from *S*_2_ to *S*_1_ upon reheating above 70 °C. The deformation of the MSMPMC was measured through image analysis and the strain was derived based on the deformation. By comparing the strains of fixed shape and programmed shape, the fixity of *S*_1_ and *S*_2_ are obtained as 96.86% and 80.19% respectively. Through comparing the strains of programmed shape and recovered shape, the recovery rate of *S*_1_ is obtained as 89.83%.

## Discussion

This study successfully demonstrates for the first time that electro-mechanical and thermo-mechanical actuation can be separately performed on a single actuator, simultaneously. These multiple inputs, which is electrical and thermal inputs, allows for more complex control than before. The underlying physics of these two actuation properties of MSMPMC have been explored. It has a sandwich structure of a thin ion-exchange membrane with noble metal chemically plated on the surface as electrodes. With a voltage applied to the surface, the free cations and water molecules migrate from the anionic binding sites to the surface cathode electrode within the membrane^44^. The electrostatic interaction results in local swelling, which leads to the bending motion, and hence, the electro-mechanical actuation. The thermo-mechanical actuation of Nafion™ results from the destabilization of electrostatic interactions between ions; meanwhile, the temporary shapes are held by the crystalline segments of Nafion™ as physical crosslinks^45^.

Based on Fig. 7, it was found that temperature had an influence on the electro-mechanical actuation effect of the MSMPMC. The resistance of the MSMPMC decreased as the temperature increased. This property could be applied on the thermal feedback of the MSMPMC. By measuring the input voltage and output current of the MSMPMC, the resistance could be derived. Based on the resistance change, the temperature of the MSMPMC actuator could be obtained.

The actuator presented above demonstrated complex 3D deformation. The bending, twisting, and oscillating motions of the actuator could be controlled simultaneously or separately by means of thermal-mechanical and electro-mechanical actuations. These two separate actuations are significant properties of the presented actuator. Based on previous work, the Nafion™ has multiple shape-memory properties and can be programmed into multiple shapes^32^ and then programmed by thermal or electric inputs^33^. One assumption is that the broad glass transition temperature could be regarded as the consecutive distribution of a series of glass transitions^46^. Within the range of the broad glass transition temperature, ~55 °C to ~130 °C, the Nafion™ could be programmed with multiple unique shapes, and recovered under different temperatures. By programming the actuator, complex shape change of the actuator could be achieved with thermal control, and the thermo-mechanical actuation could be used for overall structural deformation. Meanwhile, the MSMPMC could perform an oscillation motion by applying voltage on the surface electrodes. The actuation amplitude and frequency of the oscillation could be adjusted by changing the amplitude and frequency of input voltage. Thus, the electro-mechanical actuation of the MSMPMC could be utilized for locomotion.

In conclusion, a MSMPMC actuator with complex deformation capabilities was developed. The MSMPMC could be controlled separately by means of thermal and electrical inputs. It had the advantages of resilience and inherent softness; moreover, the electrical characteristics of the MSMPMC changed as the temperature changed. Potentially, it could be applied to medical devices and biomimetic robotics.

One potential application of MSMPMC is in underwater biomimetics, which has been studied for many years. The fish fins undergo considerable deformation, which enables the fish to generate propulsive forces and control body position. Robotic flapping foil devices were developed in order to understand the significance of flexible propulsive surfaces for locomotor performance^47^. A biomimetic fin was developed based on the monolithic fabrication of IPMC actuators^48^. Complex deformation modes can be produced. However, most of the devices contain complicated systems. MSMPMC can be used as a single actuator that performs similar deformations as does a fish fin. By programming MSMPMC to different desired shapes, and by controlling the thermal and electrical inputs, multiple degrees-of-freedom deformation of the actuator can be performed. There are three methods that can possibly be used to heat the actuator. The first method is adding another soft heating film on the surface of MSMPMC, such as Positive Temperature Coefficient (PTC) heating element. By controlling the input voltage on the heating film, the heating of the actuator can be controlled. The second method is induction heating. An additional layer of iron oxide nanoparticles will be plated on the surface of the actuator. Localized heating on the actuator can be generated by applying an alternating electromagnetic field. The third method is heating the water directly. An immersion heater with temperature feedback control will be used to heat and control the water temperature. Another potential application is the vessel catheter. Lei *et al.*^49^ developed a tube-shaped IPMC; however, bending of the tube-shaped IPMC was limited due to the stiffness of the tube. With the MSMPMC, new catheters can be fabricated with large deformations of multiple degrees of freedom, a capability that can be utilized in complex vessel networks. A flexible heating wire will be inserted inside the catheter for thermal controlling. A layer of thermal insulation film will be covered on the surface of the catheter to insulate the heat conduction between the body and the catheter.

## Methods

### Sample preparation

MSMPMC samples were prepared for the experiments. First, after roughening the surface of the Nafion™ -117 membrane sheet, the membrane was immersed in 3% hydrogen peroxide (H_2_O_2_) to eliminate organic impurities and in 1 M sulfuric acid (H_2_SO_4_) to remove the metallic impurities. Second, by immersing in a platinum complex solution (Pt(NH_3_)_4_Cl_2_·H_2_O) and then in a sodium borohydride solution (NaBH_4_), the membrane sheet was plated with the platinum metal (Pt) particles. To lower the surface resistance, the composite sheet was suspended in the Pt complex solution. Hydroxylamine hydrochloride (H_2_NOH·HCl) and hydrazine (NH_2_NH_2_·H_2_O) were added to the solution periodically. Finally, after the plating process, the sheet was soaked in a solution of lithium chloride for ion exchange. A more detailed procedure is presented by Kim *et al.*^50^.

### Experiments

**Figure 8:**
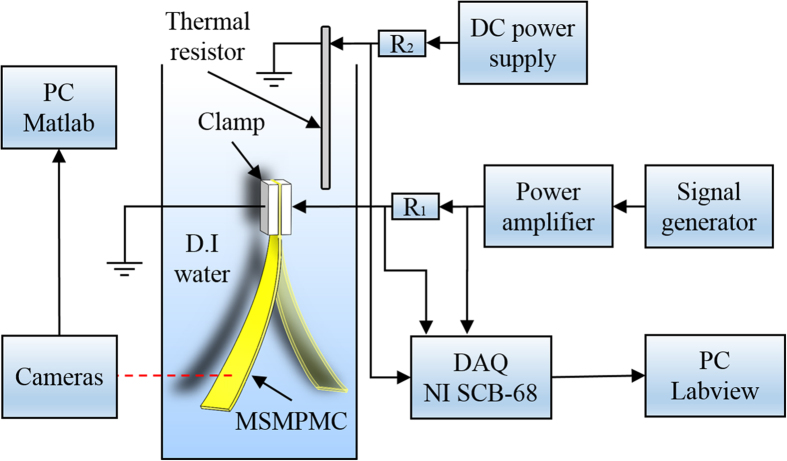
Experimental setup. The experimental setup used for measuring thermal and electromechanical responses of the MSMPMC actuator.

Figure 8 shows the schematic of the experimental setup. The MSMPMC was submerged in deionized water of 22 °C. One end of the MSMPMC was fixed by a clamp. Voltages were applied on the MSMPMC through the clamp contacts. The voltages were provided by a signal generator (FG-7002 C, EZ Digital Co., Ltd) and amplified by a power amplifier (LVC-608, AE Techron, Inc.). An immersion heater (3656K169, McMaster-Carr) was used for heating the water. A thermal resistor (PRTF-11-2-100-1/8-6-E, Omega^®^) was used to measure the water temperature. The signals were measured through DAQ (NI SCB-68, National Instruments). The input voltage, current, and temperature were recorded simultaneously, using LabVIEW 8 Software. The MSMPMC was reheated from 22 °C to 90 °C.

The radius of the curvature *ρ*_*r*_ of the MSMPMC can be denoted as^51^


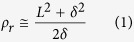


where *L* is the length and *δ* is the tip deformation of the MSMPMC. By relating the radius of the curvature *ρ*_*r*_ to strain *ε*, one can obtain


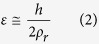


where *h* is the thickness of the MSMPMC. The fixity can be obtained by comparing the strains of fixed shape *ε*_*f*_ and programmed shape *ε*_*p*_


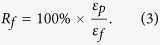


The recovery rate can be obtained by comparing the strains of programmed shape *ε*_*p*_ and recovered shape *ε*_*r*_


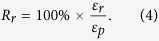


### Image analysis

To measure the 3D deformation of the MSMPMC, image analysis was used. Two cameras were set at different positions and recorded the deformation of MSMPMC during the experiments. Using an open source MATLAB^®^ program developed by Hedrick^52^, the videos from the two cameras were analyzed to measure the twisting angle and the bending deformation of the MSMPMC. Prior to imaging actuator deformation, the image analysis program was calibrated to a set coordinate frame. This was done by creating a coordinate frame specifically for the volume of space that the tank of water occupied. The videos were analyzed in the program along with the calibration coefficients file. Three points at the free end tip were tracked in the videos to determine the deformation and twist of the MSMPMC.

To calculate the bending deformation of the MSMPMC, the coordinates of the two corners *X*_*u*_, *Y*_*u*_ of the MSMPMC fixed end were measured as 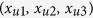 and 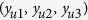. Another point *Z*_*u*_, which was in the same horizontal platform of *X*_*u*_, *Y*_*u*_ and not on the line *X*_*u*_*Y*_*u*_, was measured; the coordinate was 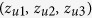. Assuming the projective point of *Z*_*u*_ on line *X*_*u*_*Y*_*u*_ is *A*_*u*_, *A*_*u*_’s coordinate, 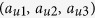 can be obtained by solving the following equations:


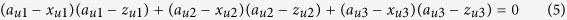



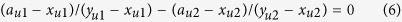



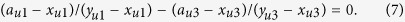


Line *X*_*u*_*Y*_*u*_ and line *Z*_*u*_*A*_*u*_ were orthogonal to each other, and line *Z*_*u*_*A*_*u*_ was in the thickness direction of the MSMPMC in a neutral position. By calculating the projective point of the MSMPMC tip point on line *Z*_*u*_*A*_*u*_, the bending displacement of the MSMPMC could be obtained. Assuming the coordinate of tip point *Z*_*d*_ was 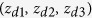, the coordinate projective point *A*_*d*_ on line *Z*_*u*_*A*_*u*_ could be obtained by using the following equations:


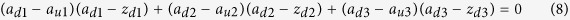



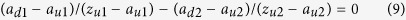



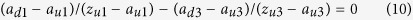


The coordinates of the two corners *X*_*u*_, *Y*_*u*_ of the tip were measured as 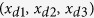 and 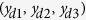. The twisting angle *θ* could be obtained by calculating the angle difference between vector *X*_*u*_*Y*_*u*_ and vector *X*_*d*_*Y*_*d*_:


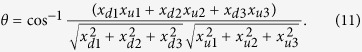


## Additional Information

**How to cite this article**: Shen, Q. *et al.* A multiple-shape memory polymer-metal composite actuator capable of programmable control, creating complex 3D motion of bending, twisting, and oscillation. *Sci. Rep.* **6**, 24462; doi: 10.1038/srep24462 (2016).

## Figures and Tables

**Figure 1:**
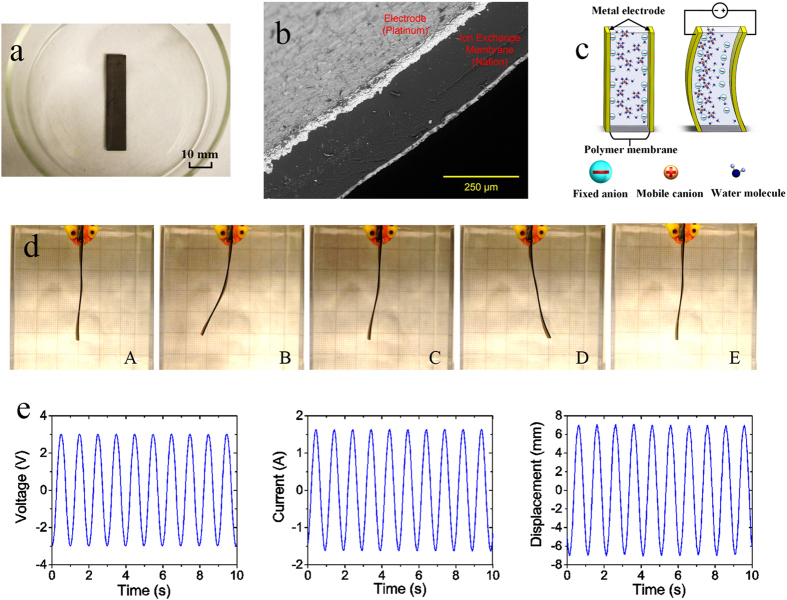
Properties of an IPMC made with Nafion™ membrane. (**a**) An IPMC sample in the evaporating pan. (**b**) A scanning electron microscopy (SEM) image of a cross-section of IPMC. The IPMC consists of the electrode on both sides and the polymer membrane between them. (**c**) An illustration of the IPMC operating principle. Deformation will occur if an electric field is applied across the IPMC, which causes the ions to redistribute along with the water molecule. The size of the IPMC is 50.78 mm in length, 9.82 mm in width and 0.53 mm in thickness. (**d**) Continuous deformation of IPMC in one cycle under the voltage of 2.6 V amplitude and 1 Hz frequency. (**e**) Input voltage, output current, and displacement of IPMC versus time under the above voltage input.

**Figure 2:**
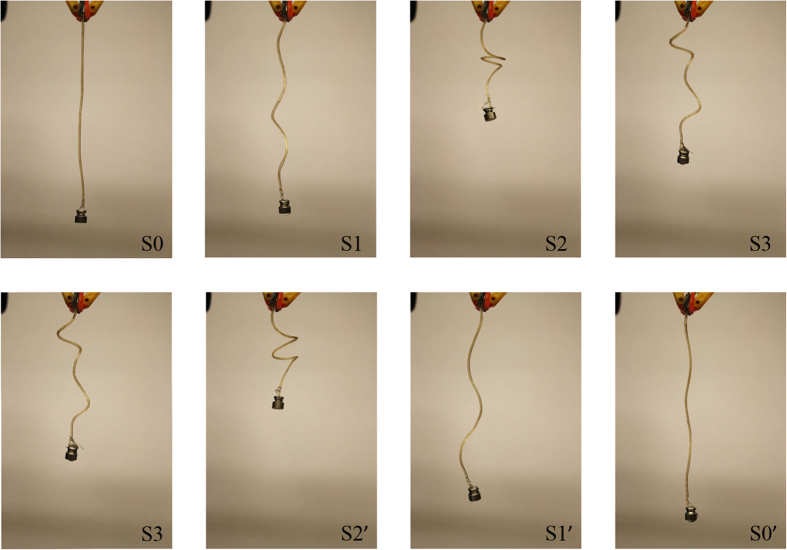
Nafion™ fiber demonstrating quadruple shape memory cycles with a 1-g weight on the tip. Triple shapes of the Nafion™ fiber, 99.87 mm in length and 0.95 mm in diameter, were programmed with loops having different shapes wrapping around a metal rod in the water. The fiber with original shape, S0, was wrapped and programmed at 85 °C and fixed at 75 °C to achieve the first programmed shape, S1. The second shape, S2, and third shape, S3, was programmed at 70 °C, 55 °C and fixed at 60 °C and 21 °C, respectively by wrapping around the rod with different cycles. Then, the Nafion™ fiber was reheated. The Nafion™ fiber recovered to S2′, S1′, and S0′ upon reheating to 55 °C, 70 °C, and 85 °C, respectively.

## References

[b1] Weihs D. Hydromechanics of Fish Schooling. Nature 241, 290–291, 10.1038/241290a0 (1973).

[b2] Engelmann J., Hanke W., Mogdans J. & Bleckmann H. Neurobiology: Hydrodynamic stimuli and the fish lateral line. Nature 408, 51–52, 10.1038/35040706 (2000). 11081502

[b3] Wilga C. D. & Lauder G. V. Biomechanics: Hydrodynamic function of the shark’s tail. Nature 430, 850, 10.1038/430850a (2004). 15318211

[b4] Nawroth J. C. *et al.* A tissue-engineered jellyfish with biomimetic propulsion. Nat. Biotechnol. 30, 792–797, 10.1038/nbt.2269 (2012). 22820316 PMC4026938

[b5] Huebsch N. & Mooney D. J. Inspiration and application in the evolution of biomaterials. Nature 462, 426–432, 10.1038/nature08601 (2009). 19940912 PMC2848528

[b6] Takashima Y. *et al.* Expansion-contraction of photoresponsive artificial muscle regulated by host-guest interactions. Nat. Commun. 3, 1270, 10.1038/ncomms2280 (2012). 23232400 PMC3535346

[b7] Sanchez C., Arribart H. & Guille M. M. G. Biomimetism and bioinspiration as tools for the design of innovative materials and systems. Nat. Mater. 4, 277–288, 10.1038/nmat1339 (2005). 15875305

[b8] Rus D. & Tolley M. T. Design, fabrication and control of soft robots. Nature 521, 467–475, 10.1038/nature14543 (2015). 26017446

[b9] Wen L., Weaver J. C. & Lauder G. V. Biomimetic shark skin: design, fabrication and hydrodynamic function. J. Exp. Biol. 217, 1656–1666, 10.1242/jeb.097097 (2014). 24829323

[b10] Shahinpoor M., Bar-Cohen Y., Simpson J. O. & Smith J. Ionic polymer-metal composites (IPMCs) as biomimetic sensors, actuators and artificial muscles - a review. Smart Mater. Struct. 7, R15–R30, 10.1088/0964-1726/7/6/001 (1998).

[b11] Egan P., Sinko R., LeDuc P. R. & Keten S. The role of mechanics in biological and bio-inspired systems. Nat. Commun. 6, 7418, 10.1038/ncomms8418 (2015). 26145480

[b12] Kim B., Lee M. G., Lee Y. P., Kim Y. & Lee G. An earthworm-like micro robot using shape memory alloy actuator. Sensors Actuators, A Phys. 125, 429–437, 10.1016/j.sna.2005.05.004 (2006).

[b13] Takashima K., Rossiter J. & Mukai T. McKibben artificial muscle using shape-memory polymer. Sensors Actuators, A Phys. 164, 116–124, 10.1016/j.sna.2010.09.010 (2010).

[b14] Shen Q., Wang T. & Kim K. J. A biomimetic underwater vehicle actuated by waves with ionic polymer–metal composite soft sensors. Bioinspir. Biomim. 10, 055007, 10.1088/1748-3190/10/5/055007 (2015). 26414228

[b15] Marchese A. D., Onal C. D. & Rus D. Autonomous Soft Robotic Fish Capable of Escape Maneuvers Using Fluidic Elastomer Actuators. Soft Robot. 1, 75–87, 10.1089/soro.2013.0009 (2014). PMC4997624 27625912

[b16] Tolley M. T. *et al.* A Resilient, Untethered Soft Robot. Soft Robot. 1, 213–223, 10.1089/soro.2014.0008 (2014).

[b17] Rolf M. & Steil J. J. Efficient exploratory learning of inverse kinematics on a bionic elephant trunk. IEEE Trans. Neural Networks Learn. Syst. 25, 1147–1160, 10.1109/TNNLS.2013.2287890 (2014).

[b18] Kurbanhusen Mustafa S., Yang G., Huat Yeo S., Lin W. & Chen M. Self-calibration of a biologically inspired 7 DOF cable-driven robotic arm. IEEE/ASME Trans. Mechatronics, 13, 66–75, 10.1109/TMECH.2007.915024 (2008).

[b19] Gupta G. S., Mukhopadhyay S. C., Messom C. H. & Demidenko S. N. Master-slave control of a teleoperated anthropomorphic robotic arm with gripping force sensing. IEEE Trans. Instrum. Meas. 55, 2136–2145, 10.1109/TIM.2006.884393 (2006).

[b20] Irschik H. A review on static and dynamic shape control of structures by piezoelectric actuation. Eng. Struct. 24, 5–11, 10.1016/S0141-0296(01)00081-5 (2002).

[b21] Pugal D., Jung K., Aabloo A. & Kim K. J. Ionic polymer-metal composite mechanoelectrical transduction: Review and perspectives. Polym. Int. 59, 279–289, 10.1002/pi.2759 (2010).

[b22] Foroughi J. *et al.* Torsional Carbon Nanotube Artificial Muscles. Science 334, 494–497, 10.1126/science.1211220 (2011). 21998253

[b23] Chopra I. Review of state of art of smart structures and integrated systems. AIAA J. 40, 2145–2187, 10.2514/2.1561 (2002).

[b24] Jo C., Pugal D., Oh I. K., Kim K. J. & Asaka K. Recent advances in ionic polymer-metal composite actuators and their modeling and applications. Prog. Polym. Sci. 38, 1037–1066, 10.1016/j.progpolymsci.2013.04.003 (2013).

[b25] Kim O., Shin T. J. & Park M. J. Fast low-voltage electroactive actuators using nanostructured polymer electrolytes. Nat. Commun. 4, 2208, 10.1038/ncomms3208 (2013). 23896756

[b26] Liu Y. *et al.* Direct observation of ion distributions near electrodes in ionic polymer actuators containing ionic liquids. Sci. Rep. 3, 973, 10.1038/srep00973 (2013). 23512124 PMC3603292

[b27] Shen Q., Kim K. J. & Wang T. Electrode of ionic polymer-metal composite sensors: Modeling and experimental investigation. J. Appl. Phys. 115, 194902, 10.1063/1.4876255 (2014).

[b28] Peraza-Hernandez E. A., Hartl D. J., Malak R. J. Jr & Lagoudas D. C. Origami-inspired active structures: a synthesis and review. Smart Mater. Struct. 23, 094001, 10.1088/0964-1726/23/9/094001 (2014).

[b29] Tanaka Y. *et al.* Ferrous polycrystalline shape-memory alloy showing huge superelasticity. Science 327, 1488–1490, 10.1126/science.1183169 (2010). 20299589

[b30] Meng H. & Li G. A review of stimuli-responsive shape memory polymer composites. Polymer. 54, 2199–2221, 10.1016/j.polymer.2013.02.023 (2013).

[b31] Felton S., Tolley M., Demaine E., Rus D. & Wood R. A method for building self-folding machines. Science 345, 644–646, 10.1126/science.1252610 (2014). 25104380

[b32] Xie T. Tunable polymer multi-shape memory effect. Nature 464, 267–270, 10.1038/nature08863 (2010). 20220846

[b33] Rossiter J., Takashima K. & Mukai T. Shape memory properties of ionic polymer – metal composites. Smart Mater. Struct. 21, 112002, 10.1088/0964-1726/21/11/112002 (2012).

[b34] Chae W., Cha Y., Peterson S. D. & Porfiri M. Flow measurement and thrust estimation of a vibrating ionic polymer metal composite. Smart Mater. Struct. 24, 095018, 10.1088/0964-1726/24/9/095018 (2015).

[b35] Shahinpoor M. Biomimetic robotic Venus flytrap (Dionaea muscipula Ellis) made with ionic polymer metal composites. Bioinspir. Biomim. 6, 046004, 10.1088/1748-3182/6/4/046004 (2011). 21992999

[b36] DeVries L., Lagor F. D., Lei H., Tan X. & Paley D. A. Distributed flow estimation and closed-loop control of an underwater vehicle with a multi-modal artificial lateral line. Bioinspir. Biomim. 10, 025002, 10.1088/1748-3190/10/2/025002 (2015). 25807584

[b37] Palmre V. *et al.* Nanothorn electrodes for ionic polymer-metal composite artificial muscles. Sci. Rep. 4, 6176, 10.1038/srep06176 (2014). 25146561 PMC4141252

[b38] Punning A. *et al.* Ionic electroactive polymer artificial muscles in space applications. Sci. Rep. 4, 6913, 10.1038/srep06913 (2014). 25372857 PMC5381380

[b39] Wu G. *et al.* Graphitic carbon nitride nanosheet electrode-based high-performance ionic actuator. Nat. Commun. 6, 7258, 10.1038/ncomms8258 (2015). 26028354 PMC4458862

[b40] Feng G.-H. & Hou S.-Y. Double-section curvature tunable functional actuator with micromachined buckle and grid wire for electricity delivery. Smart Mater. Struct. 24, 095010, 10.1088/0964-1726/24/9/095010 (2015).

[b41] Palmre V. *et al.* An IPMC-enabled bio-inspired bending/twisting fin for underwater applications. Smart Mater. Struct. 22, 014003, 10.1088/0964-1726/22/1/014003 (2013).

[b42] Xiao X. *et al.* Shape memory polymers with high and low temperature resistant properties. Sci. Rep. 5, 14137, 10.1038/srep14137 (2015). 26382318 PMC4585657

[b43] Meng Q. & Hu J. A review of shape memory polymer composites and blends. Compos. Part A Appl. Sci. Manuf. 40, 1661–1672, 10.1016/j.compositesa.2009.08.011 (2009).

[b44] Shahinpoor M. & Kim K. J. Ionic polymer-metal composites: I. Fundamentals. Smart Mater. Struct. 10, 819–833, 10.1088/0964-1726/10/4/327 (2001).

[b45] Berg G. J., McBride M. K., Wang C. & Bowman C. N. New directions in the chemistry of shape memory polymers. Polymer. 55, 5849–5872, 10.1016/j.polymer.2014.07.052 (2014).

[b46] Meng H. & Li G. A review of stimuli-responsive shape memory polymer composites. Polymer. 54, 2199–2221, 10.1016/j.polymer.2013.02.023 (2013).

[b47] Lauder G. V., Anderson E. J., Tangorra J. & Madden P. G. A. Fish biorobotics : kinematics and hydrodynamics of self-propulsion. J. Exp. Biol. 210, 2767–2780, 10.1242/jeb.000265 (2007). 17690224

[b48] Chen Z. & Tan X. Monolithic fabrication of ionic polymer-metal composite actuators capable of complex deformation. Sensors Actuators, A Phys. 157, 246–257, 10.1016/j.sna.2009.11.024 (2010).

[b49] Lei H., Sharif M. A. & Tan X. Dynamics of Omnidirectional IPMC Sensor: Experimental Characterization and Physical Modeling. IEEE/ASME Trans. Mechatronics 21, 601–612, 10.1109/TMECH.2015.2468080 (2015).

[b50] Kim K. J. & Shahinpoor M. Ionic polymer metal composites: II. Manufacturing techniques. Smart Mater. Struct. 12, 65–79, 10.1088/0964-1726/12/1/308 (2003).

[b51] Shen Q., Palmre V., Stalbaum T. & Kim K. J. A comprehensive physics-based model encompassing variable surface resistance and underlying physics of ionic polymer-metal composite actuators. J. Appl. Phys. 118, 124904, 10.1063/1.4931912 (2015).

[b52] Hedrick T. L. Software techniques for two- and three-dimensional kinematic measurements of biological and biomimetic systems. Bioinspir. Biomim. 3, 034001, 10.1088/1748-3182/3/3/034001 (2008). 18591738

